# Effects of transcutaneous electrical nerve stimulation in postoperative total knee arthroplasty pain and intravenous analgesic requirement

**DOI:** 10.1038/s41598-026-54205-9

**Published:** 2026-05-23

**Authors:** Ayesha Jamil, Ahsan Javed, Muhammad Adeel, Muhammad Akram Naseem

**Affiliations:** 1 University Institute of Physical Therapy, Faculty of Allied Health Sciences, The University of Lahore, 1-KM Defence Road, Near Bhuptian Chowk, Lahore, Punjab Pakistan; 2 Faculty of Allied Health Sciences, University of South Asia, 47-Tufail Road, Cantt, Lahore, Punjab Pakistan; 3 Master Program in Smart Healthcare Management, National Taipei University, New Taipei City, Taiwan, ROC; 4 Lahore Business School, The University of Lahore, 1-KM Defence Road, Near Bhuptian Chowk, Lahore, Punjab Pakistan

**Keywords:** Total knee arthroplasty, Transcutaneous electrical nerve stimulation, Postoperative pain, Intravenous analgesics, Diseases, Health care, Medical research

## Abstract

Total Knee Arthroplasty (TKA) is a widely used procedure to relieve disability associated with advanced knee osteoarthritis. The management of postoperative TKA is crucial for the success of surgery and patient satisfaction. This study aims to determine the effects of integrating transcutaneous electrical nerve stimulation into standard postoperative TKA care on acute resting pain scores and intravenous analgesic requirements during the first three postoperative days. This randomised controlled trial was conducted from July to December 2022 at two hospitals in Lahore, Punjab, Pakistan. A total of 60 participants with TKA, aged 41 to 85 years, were recruited through purposive sampling. The control group received standard intravenous analgesics and postoperative rehabilitation, and the experimental group received transcutaneous electrical nerve stimulation (TENS) additionally. TENS was applied at a frequency of 85 Hz, pulse width of 120 µs, and at low intensity as tolerated. Pain was measured through the numeric pain rating scale, and analgesic requirements were monitored through the prescription chart. The results showed significant between-group differences in pain reduction (*p* < 0.001), whereas no statistically significant difference was observed in drug dose (*p* = 0.06) and frequency (*p* = 0.032/ corrected *p* = 0.0167). TENS was a useful integrated modality to conservative pharmacological management of pain after total knee arthroplasty.

Trial registration: This trial was prospectively registered at ClinicalTrials.gov (Trial ID NCT05470244) on 13th July, 2022. https//clinicaltrials.gov/study/NCT05470244.

## Introduction

Total knee arthroplasty (TKA) is among the major advancements in treating pain and improving physical function in patients with knee osteoarthritis (OA)^1^. Patients with total knee arthroplasty experience moderate to severe acute pain at rest that lasts from 3 to 6 h after surgery and may continue for the subsequent 72 h, even with the provision of standard protocols and new advancements^2^. Management of acute pain at rest after TKA in the postoperative period is a big challenge to deal with and still a major concern for practitioners^3^ and patients, as it not only causes discomfort and disappointment about the procedure among the patients but also interferes with their postoperative recovery, leading to delayed ambulation^4^.

A wide variety of pharmacological modalities, including anaesthetic and analgesic agents^5^ with different routes and modes of transmission, speed up the recovery process with variable results^6^. Each pain control procedure has its limitations concerning side effects, toxicity^7^, and the monitoring process^8^. Transcutaneous electrical nerve stimulation (TENS) is a non-pharmacological, electrotherapeutic modality commonly used for alleviating pain in both acute and chronic conditions affecting the neuromusculoskeletal system of the body^9^.

TENS acts as an electroanalgesic modality by utilising neuropharmacological and pain gate theory through central and peripheral mechanisms^10^. The neuropharmacological theory of pain control explains that the release of opioids (beta-endorphins) at the point of electrical stimulation by TENS causes a reduction in pain. The pain gate theory states that the selective stimulation of mechanoreceptive nerve fibres (A beta) over nociceptive (A-delta and C-type) fibres by TENS prevents the central flow of nociceptive sensation and hence the decline in perception of pain at the site of injury^11^.

However, the literature search suggested a controversy regarding the role of transcutaneous electrical nerve stimulation^12^ and its dose^9^ in improving the functional and clinical outcomes of knee and analgesics consumption after total knee arthroplasty^11^. Therefore, this study aims to find out the effects of integrating TENS in standard postoperative TKA care on acute resting pain and intravenous analgesics requirement with the use of opioids and other analgesics given through the intravenous route in the first three postoperative days. It was hypothesized that the patients receiving TENS for post-operative management of TKA would experience a significant reduction in acute resting pain and intravenous analgesic requirements.

## Methods

This study employed a single-blind, parallel-group, randomised controlled trial design with an allocation ratio of 1:1. The trial was conducted under a superiority framework, aiming to evaluate whether the addition of Transcutaneous Electrical Nerve Stimulation (TENS) to standard postoperative care during hospitalisation following total knee arthroplasty (TKA) significantly reduces acute resting pain and the requirement for intravenous analgesics. It was conducted at the University of Lahore over a period of six months from July to December 2022. The data was collected from two private-sector hospitals in Lahore, Punjab, Pakistan. The ethical approval for the study was obtained from the Research Ethics Committee (REC) of the University of Lahore (Ref Id: REC-UOL-115-06-2022). The study adheres to the ethical principles outlined in the Declaration of Helsinki and follows the guidelines of the Consolidated Standards of Reporting Trials (CONSORT). Informed consent was taken from participants at the time of recruitment in the study. The objectives of the study and procedure were explained to them, and confidentiality of personal information and anonymity of data were ensured. The right to withdraw from the study at any time was also reserved. This trial was prospectively registered at ClinicalTrials.gov on 13/07/2022 (Trial ID: NCT05470244). No significant changes in trial protocol, outcome measures, and analysis were made after the study commenced.

### Study setting, site coordination, and staff training

The data collection was made at two hospitals, where the standardised study protocol was followed. In each hospital, there was a designated site coordinator who was responsible for patient recruitment, informed consent, intervention delivery, and outcome assessment. Both coordinators worked under the guidance of the principal investigator to maintain uniformity of procedure.

Firstly, training was given to a team of physiotherapists and nursing staff regarding the study protocols, including intervention delivery, outcome assessment, and handling adverse effects. The clinicians who performed the surgery were unaware of the group allocation and independent of the outcome assessment. The outcome assessors were instructed to follow a standardised assessment protocol to ensure consistency across both sites.

### Sample size calculation

The sample size of 60 participants was estimated using G*Power 3.1.9.7. A priori power analysis of repeated measure analysis of variance (ANOVA) for within-between interaction, using the effect size of 0.16, 5% level of significance, and 90% power of the test, 6 measurement points, and a correlation of 0.5 between measurements^11^, and adding 10% attrition rate

Effect size f = 0.16

Level of significance (α) = 0.05

Power of test (1–β) = 0.90

Number of groups = 2

Number of measurements = 6

Correlation among rep measurements = 0.5

Non-sphericity correlation = 1.

### Participant characteristics

The participants of both genders, with age ranges from 41 to 85 years, were scheduled for TKA primarily due to advanced knee osteoarthritis^13,14^. Those who reported the presence of any allergic condition of the skin, chronic consumption of opioids^15^, use of a cardiac pacemaker, major bone operation, revision knee arthroplasty^13^, neurological illness, or mental disorder that might interfere with the assessment process^14^, were excluded from the study.

### Randomisation and allocation concealment

The participants recruited through purposive sampling using a pre-defined criterion were consecutively enrolled to meet the sample size requirement. After initial screening, eligible participants were then randomly allocated into two groups using a computer-generated randomisation sequence. An independent biostatistician generated the allocation sequence in Microsoft Excel using the RANDBETWEEN(1,2) function, assigning participants to the control group (Group A) or the experimental group (Group B) in equal proportions. The control group received standard intravenous analgesic regimens and postoperative rehabilitation, while the experimental group received TENS in addition to the standard care. To ensure allocation concealment, the treatment assignments were placed in sequentially numbered, opaque, sealed envelopes by a separate researcher not involved in enrollment or intervention delivery^16^. Simple randomisation was applied without any restriction of stratification or block size.

### Interventions

#### Intravenous analgesics regimen

Intravenous analgesics, including NSAIDs such as ketorolac and/or opioids such as tramadol, prescribed by the consultant orthopaedic surgeon, were administered to participants by the ward nursing staff under the supervision of the surgical team, in both groups, as part of standard medical care for postoperative pain management. Intravenous ketorolac was given at a dose of 30 mg every 12 h or as needed, while tramadol was administered at 50–100 mg intravenously as needed, with dosing based on pain intensity reported by participants and the surgeon’s discretion.

#### Transcutaneous electrical nerve stimulation

Electrical stimulations were given in experimental group B using a TENS device of model # EV: 806, product code: Comfy Stim^®^, with a frequency of 85 Hz^17^, low intensity as tolerated by participants with no muscle contraction, and pulse width/duration of 120 microseconds. Two pairs of 40 × 40 mm-sized, self-adhesive electrode pads were placed on the painful side near the surgical incision around the knee joint or at a corresponding paravertebral dermatomal level in cases where no adjacent space was available^18^. It was applied to the participants by the trained physiotherapists with an average experience of five years in postoperative rehabilitation. The duration of TENS application was 30 min, every eight hours, for the first three postoperative days.

#### Standard postoperative rehabilitation

The standard postoperative rehabilitation protocol was given to participants of both groups. It includes ROM exercises, such as heel-sides and active/assisted knee flexion and extension, and tolerated weight-bearing with a walker under the supervision of a trained physiotherapist, starting from postoperative day 1 till day 3^19^.

### Outcome variables and assessment

Primary outcomes were acute resting pain intensity and intravenous analgesic requirement, assessed during the first three postoperative in-hospital days following total knee arthroplasty. Pain intensity was measured using the Numeric Pain Rating Scale (NPRS), an 11-point scale where “0” indicates no pain and “10” indicates the worst possible pain^20,21^. Pain scores were recorded on postoperative day 1 before the application of TENS and then twice daily from postoperative day 1 to day 3 following the application of TENS.

Analgesic requirement was determined by the patient’s pain threshold and the recommendation of the consultant orthopaedic surgeon, monitored through the prescription chart, which recorded requests for analgesics and dosages administered. Drug dosage and frequency were extracted from patient records at the end of each postoperative day (days 1 to 3). The outcome assessor was an independent musculoskeletal physiotherapist with over five years of clinical experience, blinded to the treatment groups and not involved in delivering or supervising the intervention.

### Safety considerations

Participants were regularly monitored during the hospitalisation period for any potential adverse effects related to the intervention. This included skin irritation or discomfort associated with TENS, and side effects of intravenous analgesics such as nausea or vomiting.

### Data management

The data was first entered in Microsoft Excel and then imported into the Statistical Package for the Social Sciences (SPSS) version 26 for statistical analysis. Data was stored in a password-protected electronic file that was accessible to the research team. Hard copies, such as patient consent forms and data sheets, were locked in the cabinets of the research institute. The patient’s personal information was coded with unique IDs to maintain confidentiality.

### Statistical analysis

The Statistical Package for the Social Sciences (SPSS) version 26 was used to enter and analyse data. Descriptive statistics were expressed using frequency and percentages for categorical data and mean ± standard deviation for continuous data. The normality of data was checked by the histogram, Q-Q plots, and Kolmogorov-Smirnov test (*n* ≥ 50). A mixed-design ANOVA was employed to analyse both between-group and within-group differences in mean pain scores. The Mann-Whitney U test was used to compare drug dosage between the two treatment groups, whereas within-group differences were estimated using Friedman’s ANOVA. The p-value was set at 0.05.

In addition, 2 × 3 cross-tabulation and Fisher-Freeman-Halton Exact tests were conducted to examine the association between group type and the frequency of intravenous analgesic administration, as 20% of the cells had expected frequencies less than 5. Bonferroni correction was applied to account for multiple comparisons on the three postoperative days, and statistical significance for frequency of drug administration was set at *p* < 0.0167 (0.05/3).

All participants were analysed in the groups to which they were initially assigned, following the intention-to-treat (ITT) principle. There was no loss to follow-up after randomisation; therefore, no imputation for missing data was required.

## Results

**Fig. 1:**
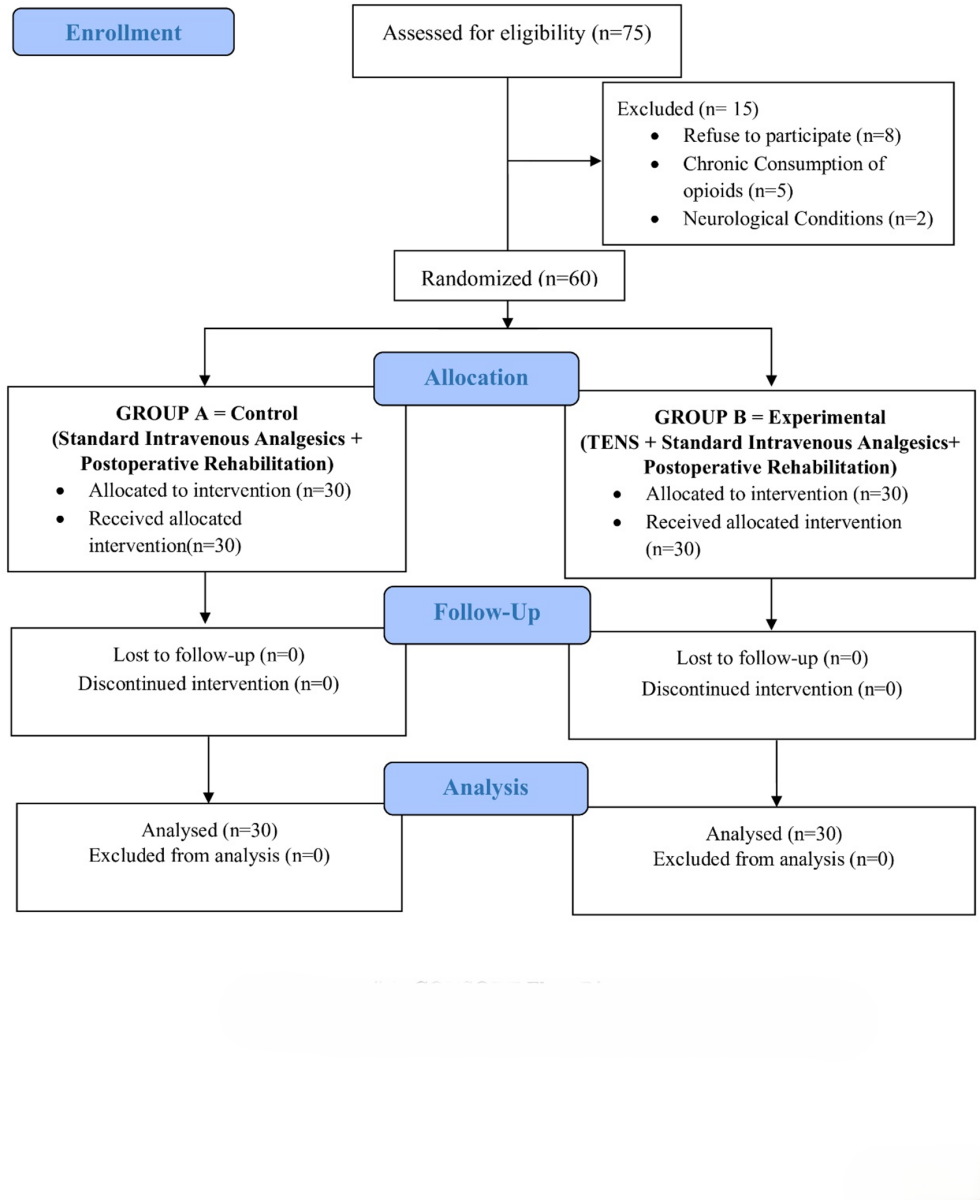
CONSORT flow-diagram.

A total of 75 individuals were assessed for eligibility, of whom 60 participants were randomised to one of the two treatment groups. There was a three-day hospitalisation period after knee arthroplasty, during which no harmful or adverse events were reported during treatment administration in any group, and no loss to follow-up. All the enrolled participants had completed the intervention, and outcomes were assessed. The process of screening, randomisation, allocation, and follow-up is summarised in the CONSORT flow diagram in Fig. 1.

The mean age of the participants was 64.96 ± 8.90 years. There were 49 (82%) females and 11 (18%) males; the majority, i.e., 44 (73%), were married, 35 (58%) belonged to a middle socio-economic status, and 36 (60%) were overweight and diagnosed with advanced osteoarthritis, resulting in TKA. The group-wise comparison of demographic and baseline characteristics is presented in Table 1, which shows that both groups are homogeneous at the start of the study (*p* ≥ 0.05).

**Table 1:** Comparison of Baseline Characteristics of Study Groups.

Study variables	Characteristics	Group A (control)	Group B (experimental)	*p*-value
		*n* (%)		
Gender	Male	5 (8)	6 (10)	0.739
	Female	25 (42)	24 (40)	
BMI	Normal	4 (7)	6 (10)	0.568
	Over-weight	20 (33)	16 (27)	
	Obese	6 (10)	8 (13)	
Marital status	Married	21 (35)	23 (38)	0.298
	Widow/widower	8 (13)	4 (7)	
	Divorced	1 (2)	3 (5)	
Monthly income	> 1,50,000 PKR	12 (20)	13 (22)	0.793
	≤ 1,50,000 PKR	18 (30)	17 (28)	
Surgical side	Right	17 (28)	18 (30)	0.793
	Left	13 (22)	12 (20)	
Comorbidities	Yes	21 (35)	17 (28)	0.284
	No	9 (15)	13 (22)	
		Mean ± SD		
Age (years)		66.50 ± 7.740	63.43 ± 9.82	0.163
Pain intensity at NRS*1		6.37 ± 0.523	6.11 ± 0.576	0.072

### Pain intensity

**Fig. 2:**
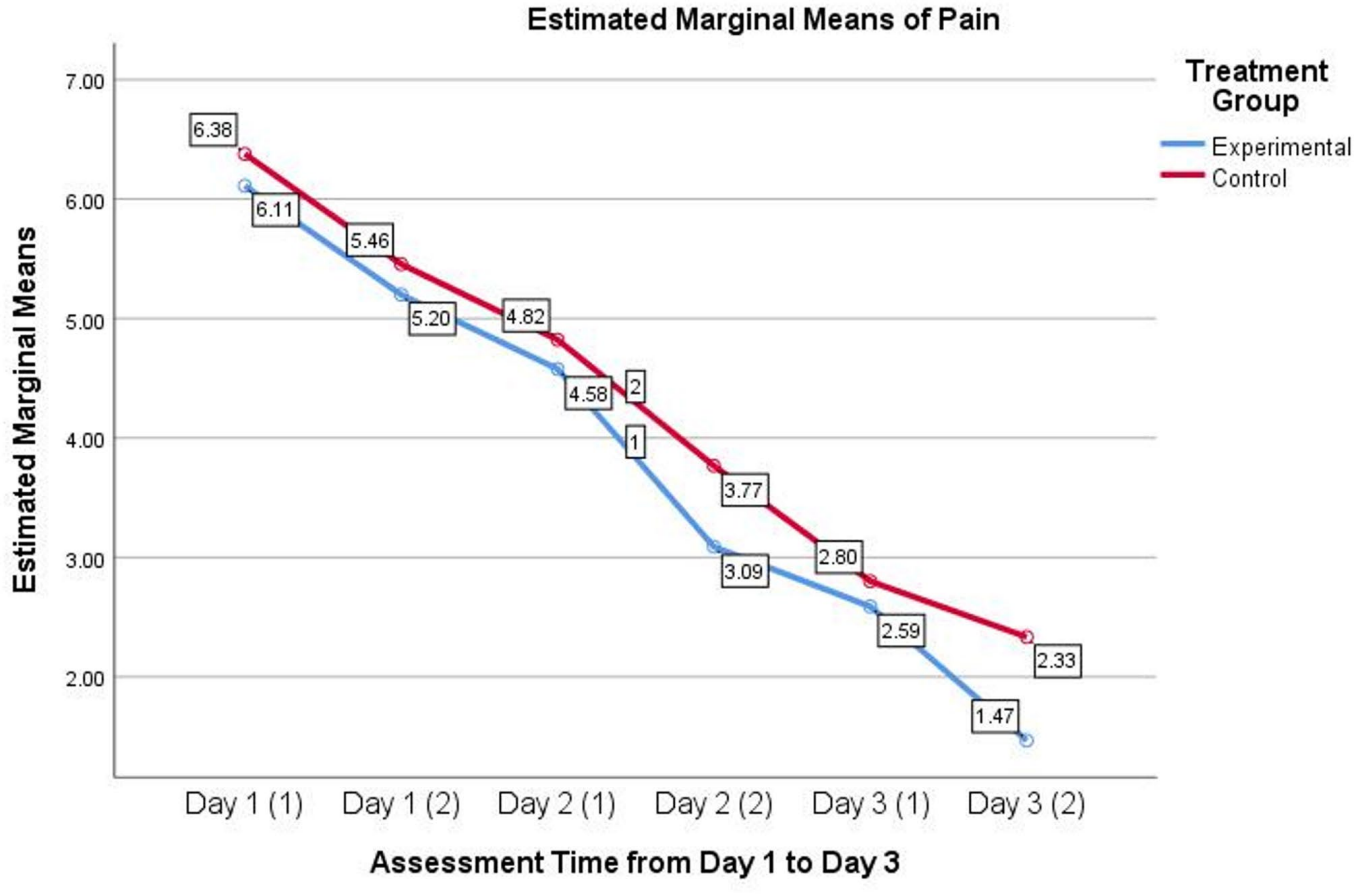
Mean pain intensity estimates across measurement times.

The mean score of pain intensity on day 1 (pre-treatment) was 6.11 ± 0.576 in the experimental group and 6.37 ± 0.523 in the control group, which was reduced to 1.46 ± 0.584 in the experimental group and 2.33 ± 0.742 in the control group by day 3, as shown in Fig. 2. The pair-wise comparison derived from mixed-design ANOVA using Bonferroni adjustment showed that the mean difference of pain score between the experimental and control group was − 0.420 [95% CI: -0.585 to -0.256], SE = 0.082, *p* < 0.001, indicating a statistically significant reduction in pain intensity in the experimental group compared to the control group.

The results of a mixed-design ANOVA to determine the pain score across time, with effects of treatment group and type of treatment intervention, are summarised in Table 2. It depicts that the pain score was significantly reduced over time for all the participants (F = 598.95, *p* < 0.001, η² = 0.912). The main effect of intervention type was also significant (F = 26.09, *p* < 0.001, η² = 0.310), showing that the experimental group experienced overall lower pain scores than the control group.

**Table 2:** Within-between group differences of pain score.

Outcome variable	Source of variations	Type III sum of squares	Mean square	F-statistics	*p*-value	Partial eta squared
Pain intensity	Time	823.08	526.97	598.95	< 0.0001	0.912
	Type of intervention	15.90	15.90	26.09	< 0.0001	0.310
	Time × type of intervention	5.87	3.76	4.27	0.025	0.069

### Intravenous analgesics dose

The Mann-Whitney U test was applied to find the between-group differences in the dose of intravenous analgesics. The results revealed that a lower dose of intravenous analgesics in the experimental group had no statistically significant difference from that in the control group from day 1 (*p* = 0.247) to day 3 (*p* = 0.189) as given in Table 3. The Friedman’s ANOVA was used to analyse the within-group difference of drug dose over the three days, and the results suggested that no statistically significant difference was observed within the two groups, showing that they had similar patterns of dose reduction as given in Table 4.

**Table 3:** Between-group comparison of intravenous analgesics dose test.

Assessment time	Treatment group	Mean rank	Sum of ranks	Mann-Whitney U	*p*-value
Day 1	Experimental	28.18	845.50	380.500	0.247
	Control	32.82	984.50		
Day 2	Experimental	27.40	822.00	357.000	0.094
	Control	33.60	1008.00		
Day 3	Experimental	28.43	853.00	388.000	0.189
	Control	32.57	977.00		

*N* = 30 in both treatment groups.

**Table 4:** Within-group comparison of intravenous analgesics dose.

Treatment group	Time of drug administration	Mean rank	Chi-square (df), *p*-value
Experimental	Intravenous analgesics dose on day 1	2.15	4.056 (2), *p* = 0.132
	Intravenous analgesics dose on day 2	1.98	
	Intravenous analgesics dose on day 3	1.87	
Control	Intravenous analgesics dose on day 1	2.10	4.789 (2), *p* = 0.091
	Intravenous analgesics dose on day 2	2.08	
	Intravenous analgesics dose on day 3	1.82	

*df*  degree of freedom.

### Frequency of intravenous analgesics

The frequency of intravenous analgesics was reduced from twice-daily (BD) to once daily (OD) over the three days. On Day 3, a higher proportion of participants in the experimental group required no analgesics, and none required a twice-daily dose, indicating better pain control. However, no statistically significant difference was observed after the Bonferroni correction of multiple comparisons at three postoperative days. The group-wise comparison of the frequency of intravenous analgesics is summarised in Table 5.

**Table 5:** Between-group comparison of the frequency of intravenous analgesics.

Measurement time	Treatment group	Frequency of intravenous analgesics			p-value*
		OD	BD	None	
Day 1	Experimental	3 (5)	8 (13.3)	19 (31.7)	0.752
	Control	4 (6.7)	10 (16.7)	16 (26.7)	
Day 2	Experimental	8 (13.3)	2 (3.3)	20 (33.3)	0.082
	Control	4 (6.7)	8 (13.3)	18 (30)	
Day 3	Experimental	4 (6.7)	0 (0)	26 (43.3)	0.032
	Control	2 (3.3)	6 (10)	22 (36.7)	

*Adjusted p-value after Bonferroni correction is 0.0167 (0.05/3).

## Discussion

Transcutaneous Electrical Nerve Stimulation (TENS) was used in this study as an adjunct to pharmacological intervention for managing postoperative pain during the first 72 h after a total knee arthroplasty procedure. It was hypothesised that TENS plays a role in reducing acute resting, postoperative pain intensity and decreasing the demand of intravenous analgesic dose and frequency, serving as a measure of recovery.

The mean age of participants in this study was 64.97 ± 8.90 years, with 82% were female. All enrolled participants with TKA were diagnosed with advanced knee osteoarthritis (OA). Most studies identified in the literature review evaluating the efficacy of TENS on postoperative TKA outcomes reported a mean participant age of 62 ± 9.5 years, with more than 50% female participants^22,23^. Similarly, the majority of participants in those studies had grade IV osteoarthritis and a pain history of six months or longer. The demographic characteristics observed in the current study are in line with previous research findings and further support the relevance and consistency of the present study’s results.

The present study utilised intravenous analgesics in combination with TENS for managing postoperative pain following TKA. In contrast, other studies have employed a variety of pharmacological agents, including central and peripheral nerve blocks, in addition to intravenous analgesics. A meta-analysis by Bjordal et al. (2003) highlighted the use of different analgesic regimens alongside TENS to achieve desired postoperative functional outcomes, reporting variable levels of effectiveness. These differences in pain management approaches may contribute to the inconsistencies observed in treatment outcomes across studies^24^.

The results of the present study suggest that TENS has a positive effect in reducing postoperative TKA pain during the first three postoperative days, with outcomes that were both clinically and statistically significant. In terms of analgesic consumption, both the dose and frequency were reduced in the experimental group compared to the control group, although these differences were not statistically significant. These findings are consistent with a study by Yongjun et al. (2017), which evaluated the efficacy of TENS based on pain intensity (VAS score), morphine consumption, and knee range of motion (ROM) within the first 24 h after surgery^25^. Their results concluded that TENS effectively reduces both pain and morphine use, contributing to improved knee function within the first postoperative day. Both studies demonstrated the beneficial effects of TENS, although with some differences. The current study assessed intravenous NSAIDs, including Toradol and tramadol (opioids) use, without measuring ROM, and extended the observation period to three postoperative days, whereas Yongjun et al. focused solely on the first 24 h.

A similar study investigating the effects of TENS on postoperative TKA rehabilitation examined outcomes such as pain, hyperalgesia, and knee function (i.e., ROM and gait) over six months. However, this study did not evaluate the impact of TENS on drug dose reduction. It concluded that the addition of TENS to pharmacological interventions significantly reduced postoperative pain during active knee extension and walking, compared to standard care, thereby supporting the findings of the current study^14^.

Similarly, a randomized controlled trial by Zhang et al. (2014) assessed the effects of TENS alongside multimodal analgesia following TKA. Their findings aligned with the present study, demonstrating that TENS not only relieved postsurgical pain but also improved knee ROM, supporting early rehabilitation. TENS was initiated 24 h postoperatively and continued for two weeks, showing fewer complications compared to multimodal analgesia administered pre-, peri-, and post-operatively^26^.

In light of the findings of this study, it is evident that TENS plays a beneficial role in reducing postoperative pain following total knee arthroplasty (TKA), a conclusion that is also well supported by existing literature. Therefore, TENS can be effectively used as an adjunct to pharmacological management after TKA, offering minimal to no side effects while facilitating the overall rehabilitation process.

However, this study had certain limitations. Firstly, sham TENS was not used in the control group, which could have balanced the 30-minute alternative treatment provided to the experimental group. Secondly, the subjective nature of pain scoring and individual differences in pain thresholds may have influenced the results, as the dose and frequency of intravenous analgesics were not standardised between the two groups. Additionally, outcome variables were assessed only for three postoperative days. Extending the follow-up period could provide further insights into the long-term effects of TENS on recovery, including range of motion (ROM), gait, and overall functional status. Moreover, details about the type of implant, anesthesia, surgical intervention, use of tourniquet, and the condition of the unoperated knee and morphine milligram equivalent (MME) should be considered in future studies to determine their impact on postoperative pain outcomes in relation to TENS.

## Conclusion

The use of TENS significantly reduced acute resting pain following total knee arthroplasty. There was a numerical reduction in the dose and frequency of intravenous analgesic use in the TENS group over the first three postoperative days compared to the control group, but that was not statistically significant. It is concluded that TENS can be considered a valuable adjunct to conventional pharmacological management for postoperative pain relief after TKA.

## Data Availability

The data are available upon reasonable request. Requests should be directed to the corresponding author at [ayeshabutt031@gmail.com](mailto: ayeshabutt031@gmail.com) . Reuse is permitted for non-commercial research with proper citation and permission.
